# Reversible infertility in male dog following prolonged treatment of *Malassezia* dermatitis with ketoconazole

**DOI:** 10.1186/s13028-021-00616-9

**Published:** 2021-11-27

**Authors:** Anna Domosławska, Sławomir Zduńczyk

**Affiliations:** Department of Animal Reproduction with Clinic, Faculty of Veterinary Medicine, University of Warmia and Mazury in Olsztyn, Oczapowskiego 14, 10-719 Olsztyn, Poland

**Keywords:** Testosterone, Semen quality, Skin lesions

## Abstract

**Background:**

Ketoconazole, an antifungal agent, adversely affects spermatogenesis in rodents, but knowledge on adverse effects of prolonged administration of ketoconazole on the fertility of male dogs is lacking. A case of reversible infertility with azoospermia in a male American Staffordshire terrier treated with ketoconazole is reported here.

**Case presentation:**

A seven-year old male American Staffordshire terrier treated for 3 months with ketoconazole for a persistent *Malassezia* dermatitis displayed reduced libido and mating of 3 bitches had been unsuccessful. The dog was presented at the clinic 40 days after the treatment had been stopped*.* At first presentation, low libido and complete absence of sperm in the ejaculate (azoospermia) associated with low testosterone level were found. Repeated examinations revealed that sperm quality and testosterone level had restored 100 days after ketoconazole had been withdrawn. Thereafter, the dog successfully mated 2 bitches*.*

**Conclusion:**

The treatment with ketoconazole for 3 months may have led to reversible infertility characterized by azoospermia. Therefore, owners of stud dogs should be informed of this risk prior to initiating such treatment and in case of infertility, previous treatment with ketoconazole should be considered as a possible cause.

## Background

Diagnosis and treatment of infertility in male dogs is becoming increasingly important in clinical practice. There is still not enough knowledge about the causes of male infertility, which can be congenital or acquired. Congenital defects are present at birth, acquired infertility appears after the animal has become fertile [1, 2]. In many cases, the cause of dog infertility remains unknown. Acquired infertility may be caused by various factors such as prostatic and testicular conditions, hormonal disturbances, infections and pharmaceutical medication [1, 2]. Many drugs such as cytotoxic agents, hormones, non-steroidal anti-inflammatory drugs, certain antibiotics, antifungal drugs and others can adversely affect male fertility through various mechanisms. These drugs may induce sexual dysfunction and spermatogenesis impairment by indirectly inhibiting testicular function or indiriectly by modification of the hypothalamic-pituitary–testicular axis [3–5]. Studies in rodents have shown that ketoconazole, an antifungal agent, can adversely impact spermatogenesis [6, 7]. To the best of the authors' knowledge, there is only one study on the effect of a single administration of ketoconazole on the semen quality in dogs [8]. There is so far no information on the effects of prolonged administration of ketoconazole on the fertility.

The aim of this study was therefore to provide a detailed evaluation of clinical and spermatological findings in a male dog with acquired infertility after long lasting ketoconazole treatment.

## Case presentation

A seven-year-old American Staffordshire terrier (body weight (BW): 31 kg) was presented for semen evaluation at the request of the owner due to acquired infertility after ketoconazole treatment for 3 months due to fungal dermatitis. Infection with *Malessezia pachydermatis* was originally diagnosed cytologically using the tape strip technique. The dog was treated in another veterinary clinic with ketoconazole (10 mg/kg BW p. o. once a day; Canizol vet, Le Vet Beheer B.V.) for one month. The dose and 1-day dosing frequency were as recommended by manufacturer. To our knowledge there was no local treatment implemented. As the dermatitis was not completely healed, the same treatment was extended for additional two months. Before treatment with ketoconazole was initiated, the dog had mated an average of 15 bitches per year with a pregnancy rate of 90% and litter size of at least five puppies. The dog had been used for natural mating or artificial insemination in different parts of the world without any observed fertility problems. There was events in the dog’s history that could explain temporary azoospermia.

Around 60 days from the beginning of the therapy, skin lesions had resolved. Then the dog was used 3 times with four days intervals as a stud dog with different females. However, none of the females became pregnant even though they were healthy and the optimal time of mating was determined using a progesterone assay. According to the owner, the mating attempts were successful but lasted much longer than before ketoconazole treatment. The owner had not been informed about adverse effects of the treatment on fertility and that the convalescence for fertility would take more than 2 months.

The dog was presented at the Department of Animal Reproduction 40 days after the treatment had been ceased. The dog was subjected to a general clinical examination, ultrasound scanning of the prostatic gland, testes and epididymis (Esoate MyLab 30 Vet Gold, microconvex probe 6.6–8.0 MHz) and semen collection. There were no pathological changes in the prostate and testes at ultrasonography. The length of the prostate was 2.1 cm, the width 2.66 cm. The length of right testis was 3.08 cm, the width 1.75 cm, the length of left testis was 3.05 cm, the width 1.93 cm.

Semen was collected by manual manipulation in the presence of a teaser bitch in heat. The male presented very low libido and it was only possible to obtain the ejaculate after 4 trials. The ejaculate was collected into pre-warmed (36–38 ºC) glass tubes with sperm fraction and prostate gland fraction collected separately (1.5 mL and 5.5 mL, respectively). Microscopy revealed azoospermia (both fractions were checked carefully as the 2^nd^ fraction was almost transparent). The procedure was repeated two days later and azoospermia was again diagnosed. Blood samples (2 mL) for hormone analysis were collected from the cephalic vein and serum analyzed for testosterone using the electrochemiluminescence immunoassay method validated for dogs (Idexx, Germany).

Further semen collections and serum testosterone measurements took place 70 and 100 days after the end of ketoconazole administration. Ejaculates were obtained without problems. Volume, concentration and motility parameters were assessed by computer assisted sperm analysis (CASA) using a Hamilton Thorne sperm analyser (IVOS 12.3). The percentage of live spermatozoa (LIVE) was estimated on dried smears stained with eosin/nigrosin. At least 200 spermatozoa were assessed using microscopy. Unstained spermatozoa (intact membrane) were classified as live and red stained spermatozoa (defect membrane) were classified as dead. For the assessment of the percentage of normal spermatozoa (NORMAL), monochromatic Diff-Quick stain was used. Two hundred spermatozoa were evaluated per slide, representing 100%.

On day 70 sperm quality parameters, especially the concentration, percentage of progressive motile spermatozooa and percentage of abnormal spermatozooa, were poorer than reported for fertile dogs. Therefore it was not recommended to use the dog for mating. After one further month parameters of semen quality were within the physiological range (Table 1).

**Table 1:** Semen parameters on days 70 and 100 after the end of ketoconazole administration

Parameter	70 days after ketoconazole administration	100 days after ketoconazole administration	Reference values [9, 10]
Volume (mL)	3.0	4.5	3.9 ± 1.2
Concentration (× 10^6^/mL)	105	295	292.6 ± 208.3
TSC (× 10^6^)	315	1327.5	707.6 ± 498.7 1200
MOT (%)	68	95.0	88.3 ± 18.4
PMOT (%)	50	74	60–70
VAP (μm/s)	147.7	157.6	124.3 ± 19.7
VSL (μm/s)	138.5	146.6	113.0 ± 20.2
VCL (μm/s)	188.2	201.6	160.7 ± 19.7
ALH (μm)	3.9	4.1	5.0 ± 0.7
BCF (Hz)	27.8	35.7	26.2 ± 4.4
STR (%)	92	92	88.9 ± 3.4
LIN (%)	69	69	70.1 ± 7.5
RAPID (%)	53	79	65.2 ± 21.7
MEDIUM (%)	15	16	3.4 ± 2.4
SLOW (%)	21	4	19.7 ± 12.8
STATIC (%)	11	1	11.8 ± 14.4
NORMAL (%)	69	88	63.3 ± 28.5
LIVE (%)	77	93	86.8 ± 10.4

*TSC* Total Sperm Count, *MOT* motile spermatozoa, *PMOT* progressive motility, *VAP* velocity average pathway, *VSL* velocity straight line, *VCL* velocity curvilinear, *ALH* amplitude lateral head, *BCF* beat cross frequency, *STR* straightness, *LIN* linearity, motility subcategories: RAPID, MEDIUM, SLOW, STATIC

Seventy days after the end of ketoconazole administration the testosterone value in serum had returned to the physiological range (Table 2).

**Table 2:** Testosterone levels in serum on days 40, 70 and 100 after the end of ketoconazole administration

Testosterone ng/mL			
At 1st presentation	70 days after the end of ketoconazole administration	100 days after the end of ketoconazole administration	Reference values
40	70	100	
< 0.05	4.5	4.9	> 0. 3

Additional USG measurements of the prostate and testes were assessed on the day of the last semen collection. The size of the prostate didn’t change significantly, and the length was 2.7 cm. The length of right testis was 3.4 cm, the width 1.95, the length of left testis was 3.25 cm, the width 2.15 cm.

The male mated 2 females after 105 and 110 days from the end of ketoconazole treatment and they delivered 5 and 7 live puppies, respectively.

## Discussion and conclusions

Ketoconazole is widely used to treat fungal diseases in companion animals. The drug is a synthetic, broad-spectrum imidazole that exerts an antifungal effect by a mechanism that causes increased membrane permeability, inhibits uptake of precursors of RNA and DNA and synthesis of oxidative and peroxidative enzymes. However, ketoconazole inhibits the action of the cytochrome P450 enzymes involved in steroidogenesis and thus reduces the synthesis of testosterone [11, 12]. Ketoconazole is a potent inhibitor of testosterone synthesis in isolated rat Leydig cells [13] and in dispersed human, dog and rat testicular cells [14]. It has been reported that ketoconazole significantly suppressed testosterone concentrations in dogs [8, 15], humans [13, 16], rats [6] and rabbits [17]. Studies in rodents showed that ketoconazole also impairs spermatogenesis, leading to decreased semen quality. These effects are reversible [6, 7]. In our case, the dog was treated with ketoconazole in another veterinary clinic, and the therapy lasted 3 months to clinical and mycological cure of the dermatitis, but the prolonged therapy resulted in infertility and azoospermia. In most cases the length of treatment for *Malassezia* dermatitis varies between three and four weeks [18]. However, in some cases the duration of treatment is up to 300 days [19]. In accordance with the recommendations of the ketoconazole manufacturer, therapy of *Malassezia* dermatitis should be continued for an adequate period of time to ensure mycological cure. If lesions persist after 8 weeks of treatment, medication should be re-evaluated. Ketoconazole can cause adverse effects in 15% of dogs, most commonly gastrointestinal conditions, but without correlation between adverse effects and duration of therapy [19]. In our case, no gastrointestinal adverse effects such as diarrhoea, vomiting, reduced appetite, were observed.

In infertile dogs, semen should be re-evaluated 62 to 70 days after treatment with ketoconazole, i.e., after a spermatogenic cycle [1]. In the reported case, the spermatogenesis was restored 70 days after ending theketoconazole treatment. The semen quality was still poor at day 100 although being within physiological range. Similar development of semen quality was observed following reversible downregulation of testicular function in male dogs with a GnRH agonist implant [20, 21].

So far, little is known about the effect of ketoconazole on canine semen parameters and testosterone level. Vickery et al. [8] showed that single administration of ketoconazole in Beagle dogs was associated with a decline in sperm motility. Plasma testosterone decreased below 1 ng/mL and returned to normal values after 72 h. De Coster et al. [15] and Willard et al. [22] also reported a significant decrease in blood plasma testosterone concentration after administration of ketoconazole in male dogs. In vivo perfusion of canine testes with ketoconazole inhibited the stimulation of testosterone production by human chorionic gonadotropin in a dose-dependent manner [23].

In our case, the administration of ketoconazole for 3 months resulted in low testosterone concentration in blood plasma, decreased libido and complete absence of spermatozoa in the ejaculate (azoospermia). Prostate and testes size were smaller than reported for medium-sized dogs [24, 25]. However, the prostatic and testicular parenchyma was homogenous. Testicular degeneration is usually associated with a reduction of testes size and poor semen quality, but the ultrasound appearance of their parenchyma is not always altered. The testicular parenchyma of atrophic testes was described as hypoechoic to isoechoic (normal) [26]. England [27] found that poor semen quality was often associated with testes which appeared more heterogenous.

Azoospermia, lack of spermatozoa in the ejaculate, is mainly caused by failure of spermatogenesis (non-obstructive azoospermia), incomplete ejaculation or bilateral outflow obstruction (obstructive azoospermia). Both conditions can be distinguished by determination of alkaline phosphatase in seminal plasma, however, it was not determined in the present case. The possibility of an azoospermic ejaculate caused by incomplete ejaculation due to low libido cannot be excluded [28]. In our case azoospermia seems be related to inhibition of spermatogenesis due to low testosterone level. Testosterone is secreted in pulses, which may contribute to different values. However, taking into account reduced testes size, low libido and azoospermia, it can be assumed that the level of this hormone at the first presentation was really low due to long-term administration of the drug.

Testosterone plays an essential role in maintaining spermatogenesis [29]. Downregulation of endocrine testicular function (testosterone < 0.1 ng/mL) with gonadotropin-releasing hormone agonist implant in male dogs led to arrest of spermatogenesis and recrudescence of spermatogenesis was related to an increase of testosterone [20, 21]. Ketoconazole may also directly affect germ cells [30]. Orally administered ketoconazole was found in seminal plasma trough 30 h after dosing [8]. It has been reported that ketoconazole induced degeneration of the seminiferous tubules and arrested spermatogenesis in testes of rats [31] and rabbits [17].

The exact cause of acquired infertility in male dog is difficult to diagnose and remains unresolved in about 50% of cases [32]. A very detailed history of the general health and reproductive history, general and genital clinical examination, semen collection and evaluation, and depending on the case, complementary examinations (e.g., hormonal assays) are necessary to address the dog's fertility problem [1]. Since many drugs can affect sperm quality, current and previous medications should be obligatory considered.

In conclusion, it seems that prolonged treatment with ketoconazole may lead to reversible infertility characterized by azoospermia in male dogs. Therefore, owners of stud dogs should be informed of this risk prior to initiating such treatment and in a case of infertility, previous treatment with ketoconazole should be considered as a possible cause.

## Data Availability

The datasets used and analyzed in the current study are available from the corresponding author on reasonable request.

## Abbreviations

BW: Body weight
CASA: Computer-assisted semen analysis
TSC: Total sperm count
MOT: Total motility
PMOT: Progressive motility
VAP: Velocity average pathway
VSL: Velocity straight line
VCL: Velocity curvilinear
ALH: Amplitude lateral head
BCF: Beat cross frequency
STR: Straightness
LIN: Linearity
